# The DADYS-Screen: Development and Evaluation of a Screening Tool for Affective Dysregulation in Children

**DOI:** 10.1177/10731911221082709

**Published:** 2022-03-18

**Authors:** Christiane Otto, Anne Kaman, Claus Barkmann, Manfred Döpfner, Anja Görtz-Dorten, Claudia Ginsberg, Sara Zaplana Labarga, Anne-Katrin Treier, Veit Roessner, Charlotte Hanisch, Michael Koelch, Tobias Banaschewski, Ulrike Ravens-Sieberer

**Affiliations:** 1 University Medical Center Hamburg-Eppendorf, Germany; 2 University of Cologne, Germany; 3 TU Dresden, Germany; 4 University of Ulm, Germany; 5 Rostock University Medical Center, Germany; 6 University of Heidelberg, Mannheim, Germany

**Keywords:** affective dysregulation, irritability, children, screening, parent-report, item response theory

## Abstract

Affective dysregulation (AD) in children is characterized by persistent irritability and severe temper outbursts. This study developed and evaluated a screening questionnaire for AD in children. The development included the generation of an initial item pool from existing instruments, a Delphi rating of experts, focus groups with experts and parents, and psychometric analyses of clinical and population-based samples. Based on data of a large community-based study, the final screening questionnaire was developed (*n* = 771; 49.7 % female; age *M* = 10.02 years; *SD* = 1.34) and evaluated (*n* = 8,974; 48.7 % female; age *M* = 10.00 years; *SD* = 1.38) with methods from classical test theory and item response theory. The developed DADYS-Screen (*D*iagnostic Tool for *A*ffective *Dys*regulation in Children—*Screen*ing Questionnaire) includes 12 items with good psychometric properties and scale characteristics including a good fit to a one-factorial model in comparison to the baseline model, although only a “mediocre” fit according to the root mean square error of approximation (RMSEA). Results could be confirmed using a second and larger data set. Overall, the DADYS-Screen is able to identify children with AD, although it needs further investigation using clinical data.

Affective dysregulation (AD) is characterized by persistent irritable mood and severe outbursts of temper, which are among the most common and challenging symptoms in child and adolescent psychiatry (Baweja et al., 2016). In the literature, the terms AD and irritability are largely used as synonyms (Döpfner et al., 2019; Giller et al., 2021; Waltereit et al., 2019). Children with AD or irritability are excessively angry and aggressive in response to negative emotional stimuli. AD is often characterized as a transdiagnostic dimension (Döpfner et al., 2019) and is associated with a wide range of internalizing and externalizing mental disorders, including depression, anxiety, attention-deficit hyperactivity disorder (ADHD), oppositional defiant disorder (Roy et al., 2013), and conduct disorder (Axelson et al., 2012; Brotman et al., 2006; Copeland et al., 2013; Roy et al., 2013), which can result in poor diagnostic specificity (Holtmann et al., 2017).

In recent years, there has been a controversial debate about the diagnostic classification of children with AD (Grimmer et al., 2010; Parens & Johnston, 2010). As a consequence of this debate, a new diagnosis for children was introduced in the revision of the *Diagnostic and Statistical Manual of Mental Disorders* (5th ed.; *DSM-5*; American Psychiatric Association, 2013), called disruptive mood dysregulation disorder (DMDD). With an onset usually before the age of 10 years, DMDD has two core symptom criteria: severe, recurrent temper outbursts and persistent irritability. Considering the lack of empirical studies on DMDD, the inclusion of this new diagnosis has been controversially discussed (Birkle et al., 2017). In line with the proposal of Lochman et al. (2015), in the recently published 11th revision of the International Classification of Diseases (ICD-11), AD was introduced not as an independent diagnosis but as a specifier for the existing diagnosis of the oppositional defiant disorder, to differentiate between oppositional defiant disorder with and without chronic irritability and anger (World Health Organization, 2019). As the symptomatology of AD cuts across multiple diagnoses, AD fits well within the research framework RDoC (Research Domain Criteria) by the National Institute of Mental Health—a transdiagnostic dimensional approach to understand mental disorders (Insel et al., 2010). The RDoC concept encompasses the construct of frustrative nonreward, which is defined by reactions elicited in response to the omission of an expected reward and into which children with AD can be classified (Meyers et al., 2017; Morris & Cuthbert, 2012).

Despite increasing interest among researchers and clinicians, relatively little empirical research has yet been performed on the assessment, etiology, and epidemiology of AD. Depending on the underlying conceptualization of AD, previous epidemiological studies have reported prevalence rates between 0.8% and 9.2% (Brotman et al., 2006; Copeland et al., 2013; Grau et al., 2018; Mayes et al., 2015). Affected children are severely impaired in various life domains, such as school and family life (Copeland et al., 2013; Dougherty et al., 2014). Furthermore, AD in childhood predicts later psychiatric problems such as depression, anxiety, and suicidal ideation (Benarous et al., 2019; Stringaris et al., 2009; Vidal-Ribas et al., 2016) and is a common cause of health services utilization (Dougherty et al., 2016), pointing out a significant public health impact. Overall, these findings underline the importance to identify and treat children with AD at an early stage.

So far, however, knowledge regarding the appropriate assessment of AD in children is still limited. Some standardized questionnaires and structured clinical interviews include items that measure certain aspects of AD, such as irritability (Affective Reactivity Index; Stringaris et al., 2012), anger (PROMIS Anger Scale; Irwin et al., 2012), emotion regulation (Emotion Regulation Checklist; Shields & Cicchetti, 1997), or temper loss (Multidimensional Assessment of Preschool Disruptive Behavior; Wakschlag et al., 2012). Further established rating scales assessing emotion dysregulation include the Emotion Dysregulation Inventory (Mazefsky et al., 2018) and the dysregulation profiles of the Strengths and Difficulties Questionnaire (SDQ-DP; Deutz et al., 2018) and of the Child Behaviour Checklist (CBCL-DP; Geeraerts et al., 2015). However, most existing instruments are not structured around the phenotype of AD, as they do not describe the full spectrum of irritability and temper outbursts or cover additional symptoms that rather capture other disorders (McTate & Leffler, 2017; Waltereit et al., 2019). The development of focused screening and assessment tools to guide diagnosis and treatment for AD should be the focus of future research.

The aim of this study was to develop and evaluate a parent-reported screening questionnaire on AD in 8- to 12-year-old children following a mixed-methods approach. We aimed to investigate the following research questions.

**Research Question 1:** Which items from existing measures can be used to create a corresponding item pool for measuring AD?
**Research Question 2:** Which items out of this item pool should be used in a preliminary version of a screening tool on AD due to evaluations of clinical experts (Delphi ratings and focus group), ratings of parents (focus groups) and according to clinical and archived population-based data (based on methods of classical test theory [CTT])?
**Research Question 3:** Which items out of the preliminary version of the screening tool are psychometrically sound, valid, and reliable to measure AD (based on methods of CTT and item response theory [IRT]) according to prospectively gathered population-based data and should be included in the final screening tool?
**Research Question 4:** How valid and reliable is the final screening tool on AD (based on CTT and IRT) according to a large sample with prospectively gathered population-based data?

## Method

### Study and Samples

The research consortium ADOPT (*A*ffective *D*ysregulation—*O*ptimizing *P*revention and *T*reatment) aims at developing valid assessment tools for AD, investigating the epidemiology of AD and developing and evaluating treatment approaches for affected children and their parents. For more information on the consortium ADOPT including a description of the design, methods, and all included subprojects, see Döpfner et al. (2019). The tasks of the subproject ADOPT Epidemiology are to develop, evaluate, and validate a screening tool on AD assessing and using data of a large community sample. ADOPT Epidemiology was approved by the ethics committee of the General Medical Council Hamburg and the commissioner for data protection from the University Hospital Cologne.

For developing the preliminary version of the screening questionnaire (i.e., pre-DADYS-Screen), two samples were used.

- Sample 1: The clinical pre-DADYS-Screen development sample included data from a clinical pilot study conducted at the outpatient clinic of the School of Child and Adolescent Cognitive Behavior Therapy (AKiP) at the Department of Child and Adolescent Psychiatry, Psychosomatics and Psychotherapy at the University of Cologne, Germany; the clinical sample was recruited during the usual clinical assessment meetings, during which parents were informed about the study and asked to fill out a paper-and-pencil questionnaire. Data were gathered over 4 months (September–December 2017) from parents of *n* = 141 children aged 8 to 12 years referred for treatment (28 % female; *M*_age_ = 9.90 years, *SD*_age_ = 1.35).
- Sample 2: The population-based pre-DADYS-Screen development sample resulted from the longitudinal BELLA study on mental health and well-being in children and adolescents in Germany (Ravens-Sieberer et al., 2015); parent-reported data from the second measurement point of the BELLA study (conducted 2004 to 2007) on 8- to 12-year-olds were used (*n =* 1,089; 46 % female; *M*_age_ = 9.96 years, *SD*_age_ = 1.38).

For developing and evaluating the final version of the screening questionnaire (i.e., DADYS-Screen), we prospectively recruited a large community sample in the project ADOPT Epidemiology. Data were collected across four German cities (Cologne, Dresden, Mannheim, and Ulm) over 18 months (February 2018 to August 2019). Families with children aged 8 to 12 years were randomly selected from the official registers of the residents’ registration offices. Potential participants were contacted by the ADOPT Epidemiology study team using conventional mail to inform about the study and ask for written informed consent to participate. In addition, the screening questionnaire was sent out to the parents. Parents were reminded once within the study (after 4 weeks). Alternative to the paper-and-pencil questionnaire, participants had the opportunity to fill in the questionnaire online or to answer the questions on the phone. Data collection and management were supported using a secure, web-based application named REDCap (Harris et al., 2009) hosted at the Clinical Trials Center Cologne.

Overall, we contacted *n* = 79,015 parents of children aged 8 to 12 years in the abovementioned cities out of which 5% (*n* = 3,897) could not be reached by the given addresses. Out of the remaining families (*n* = 75,118), *n* = 10,288 parents gave their informed consent and participated in the study (response rate: 14 %), and 1 % of the families (*n* = 1,060) actively refused participation (main reasons for refusal, if mentioned, were “no interest” and “no time”). We had to exclude participants from the overall study sample due to (a) children who did not fit the relevant age range anymore (*n* = 194), (b) inconsistent information on age and/or gender of the respective child (*n* = 236), and (c) missing responses to all items of the screening tool (*n* = 99). This resulted in a total sample of *n* = 9,759 parents of children aged 8 to 12 years who participated in the study and responded to at least 1 item of the screening questionnaire.

For developing and evaluating the final version of the DADYS-Screen, the following samples from the community-based study ADOPT Epidemiology were used:

- Sample 3: The community DADYS-Screen development sample included valid data of the first *n* = 771 participants gathered in the project ADOPT Epidemiology until the end of March 2018 with the preliminary version of the screening tool (i.e., pre-DADYS-Screen).
- Sample 4: The community DADYS-Screen evaluation sample included data of *n* = 8,988 participants who participated from April 2018 until August 2019 in the project ADOPT Epidemiology.

**Figure 1:**
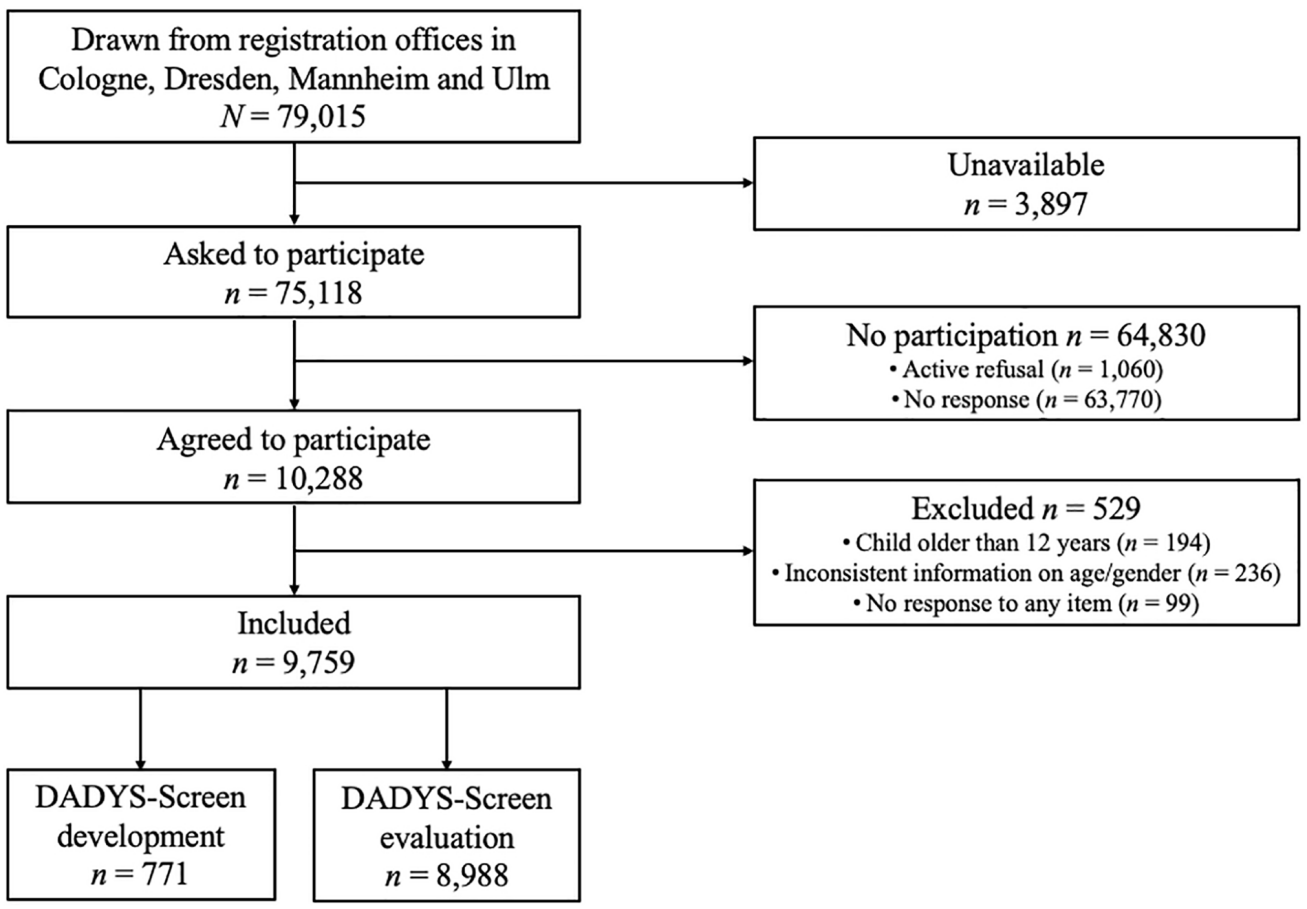
Selection of Study Participants in the Community Sample *Note.* DADYS-Screen = *D*iagnostic Tool for *A*ffective *Dys*regulation in Children—*Screen*ing.

The flowchart on the process of selecting the study participants in the community-based study is presented in Figure 1. In addition, both subsamples (Samples 3 and 4) and the total sample from the community-based study (gathering Samples 3 and 4) are described in Table 1.

**Table 1.** Characteristics of the Samples Used for Developing and Evaluating the DADYS-Screen, and for the Total Community Sample.

Characteristic	Community DADYS-Screen development sample (Sample 3: *n* = 771)	Community DADYS-Screen evaluation sample (Sample 4: *n* = 8,988)	Total community sample (Samples 3 and 4: *n* = 9,759)
Age, *M* (*SD*)	10.02 (1.34)	10.00 (1.34)	10.00 (1.38)
Female, *n* (%)	383 (49.7)	4,373 (48.7)	4,756 (48.7)
Respondent, *n* (%)			
Mother	614 (79.6)	6,889 (76.8)	7,513 (77.0)
Father	125 (16.2)	1,547 (17.2)	1,672 (17.1)
Mother and father	25 (3.2)	457 (5.1)	482 (4.9)
Grand-/step-/adoptive/foster parents or other caregivers	7 (0.9)	85 (0.9)	92 (0.9)
Assessment mode, *n* (%)			
Paper-pencil	574 (74.4)	6,810 (75.8)	7,384 (75.7)
Online	196 (25.4)	2,168 (24.1)	2,364 (24.2)
Telephone	1 (0.1)	10 (0.1)	11 (0.1)
Educational level of parents, *n* (%)			
Low	24 (3.1)	289 (3.2)	313 (3.2)
Medium	204 (26.5)	2,395 (26.6)	2,599 (26.6)
High	510 (66.1)	6,026 (67.0)	6,536 (67.0)
Missing	33 (4.3)	278 (3.1)	311 (3.2)
General health state, *n* (%)			
Very good	519 (67.3)	5,912 (65.8)	6,431 (65.9)
Good	222 (28.8)	2,748 (30.6)	2,970 (30.4)
Moderate	22 (2.9)	256 (2.8)	278 (2.8)
Bad/very bad	5 (0.6)	31 (0.4)	36 (0.4)
Missing	3 (0.4)	41 (0.5)	44 (0.5)

*Note.* Data were assessed from February to March 2018 for the community DADYS-Screen development sample and from April 2018 to August 2019 for the community DADYS-Screen evaluation sample; the samples are described in further detail in the manuscript (see Methods/Study and Samples). DADYS-Screen = *D*iagnostic Tool for *A*ffective *Dys*regulation in Children—*Screen*ing.

### Measurement

In the community samples of the project ADOPT Epidemiology, the preliminary version of the screening tool with 14 items (i.e., pre-DADYS-Screen) was administered in Sample 3 until March 2018, and the final version with 12 items (i.e., DADYS-Screen) was administered in Sample 4 from April 2018 until August 2019. For assessing data in the community study, the items for pre-DADYS-Screen and for DADYS-Screen were consistently presented with a 4-point response scale, ranging from 0 = *not at all (true)* to 3 = *especially/very (true)*. Throughout the data assessment, parents further responded to sociodemographic questions. Parental education was assessed by 2 items asking for the highest academic and vocational qualifications of both parents. According to the international “Comparative Analysis of Social Mobility in Industrial Nations” (CASMIN) classification (Brauns et al., 2003), a categorization into parents with low, medium, and high education was performed. Furthermore, general health of the child was assessed using the item “In general, how would you rate your child’s health?” provided with a 5-point scale (1 = *excellent* to 5 = *poor*).

### Analyses

#### Development of DADYS-Screen

The development of the screening tool followed a stepwise process using qualitative and quantitative methods.

##### Generation of the Initial Item Pool

The initial item pool was created based on existing instruments. Clinical and research experts reviewed well-established, validated, and standardized clinical rating scales and selected items on symptoms and behaviors closely related to AD (such as irritable mood, emotion dysregulation, anger, impulsivity, and reactivity). The following measures were selected: (a) the Affective Reactivity Index (ARI), which is a dimensional measure of irritability and anger that includes six items on behaviors and feelings specific for irritability as well as 1 item on impairment (Stringaris et al., 2012); (b) the Emotion Regulation Checklist (ERC), which is a 24-item scale for assessing the ability of a child to manage and cope with emotions (Shields & Cicchetti, 1997); (c) the PROMIS Anger Scale, which consists of 5 items covering parent-reported irritable and angry mood of the child (Irwin et al., 2012); (d) the Global Index of the Conners’ Rating Scale, which is used to screen for symptoms of ADHD and behavioral problems, and consists of ten items (Conners et al., 2011; Otto et al., 2018); (e) the dysregulation profile of the SDQ, which assesses affective, behavioral, and cognitive aspects of dysregulation (Deutz et al., 2018); and (f) the Disruptive Mood Dysregulation and Irritability Scale from the Symptom-Checklist for Oppositional Defiant Disorder and Conduct Disorder (SCL-ODD), which assesses the symptom criteria of DMDD according to *DSM-5* and is part of the German Diagnostic System for Mental Disorders in Children and Adolescents (DISYPS-III; Döpfner & Görtz-Dorten, 2017).

##### Qualitative and Quantitative Investigations of the Initial Item Pool to Develop Pre-DADYS-Screen

Qualitative investigations included a Delphi rating and focus groups. The Delphi process is a consensus method frequently used in health research with the aim to determine the extent to which experts agree on a given issue (Jones & Hunter, 1995; Landeta, 2006). In this study, a multidisciplinary group of experts, composed of child and adolescent psychologists, psychotherapists, pediatricians, and researchers, was asked to participate because of their expertise and clinical experience in the field of child mental health (*n* = 12 experts were invited, *n* = 8 consented to participate). In the first round of the Delphi rating, experts were asked to define the construct of AD. Content analysis was used to categorize responses and to develop a definition of AD as a conceptual basis. In the second round, experts were asked to evaluate the joint definition of AD. In the third round, experts were presented with the complete initial item pool and asked for their level of agreement with the inclusion of each item based on a 4-point response scale ranging from 0 (*strongly disagree*) to 3 (*strongly agree*). The positive consensus was reached if at least 90% of the experts agreed with the inclusion (*rather agree* and *strongly agree*). A negative consensus was reached if only 10% or less of the experts agreed with the inclusion.

Focus groups were conducted with (a) clinical experts (*n* = 9 clinicians and psychotherapists) working at the AKiP outpatient clinic at the Department of Child and Adolescent Psychiatry, Psychosomatics and Psychotherapy at the University of Cologne with 4 to 20 years of training and experience in the field of clinical diagnostics and treatment of children with mood and behavior problems and with (b) parents of children (*n* = 11) who were receiving psychological services due to symptoms of irritability and temper problems at the AKiP outpatient clinic at the Department of Child and Adolescent Psychiatry, Psychosomatics and Psychotherapy at the University of Cologne by trained moderators. In the first focus group, clinical experts evaluated the relevance and comprehensiveness of all items included in the initial item pool. Clinical experts discussed which items are most important in assessing AD and which items are redundant or rather capture other clinical symptoms. In the second focus group, parents were asked to comment on the comprehensibility of the items. Both focus groups were audio-recorded, transcribed verbatim, and content analyzed.

For the quantitative investigation of the initial item pool, Sample 1 (i.e., the clinical pre-DADYS-Screen development sample resulting from the clinical pilot study; *n* = 141) and Sample 2 (i.e., the population-based pre-DADYS-Screen development sample resulting from the BELLA study; *n* = 1,089) were used. Analyses based on methods from CTT were conducted. We investigated item distributions, item difficulties, and factor loadings (resulting from exploratory factor analysis, i.e., a principal axis analysis), and corrected item-total correlations. Please note, positively phrased items were recoded prior to conducting exploratory factor analyses.

Based on the results of quantitative and qualitative investigations, the initial item pool was reduced following a stepwise procedure. In the first step, we excluded items that did not meet a priori defined criteria. An item was excluded, if (a) only 10% or less of the experts agreed with the inclusion (resulting from the Delphi rating), (b) only 10% or less of the experts rated the item as relevant (resulting from the clinician focus group), (c) the item was difficult to comprehend by parents (resulting from the parent focus groups), (d) the item had a low item difficulty (only relevant for Sample 1; *P*_i_ < .20 for negatively and *P*_i_ > .80 for positively phrased items), (e) the item had a low factor loading (*a*_i1_ < .30), and/or (f) the item had a low corrected item-total-correlation, *r*_i(t-i)_ < .30. In a second step, the remaining items were reviewed and discussed in a group of six clinical and research experts. If 2 or more items were very similar in content, the experts decided which item to exclude considering qualitative and quantitative results. In addition, items that did not match the relevant content of the AD construct (low content validity) were excluded based on a final agreement among all experts. This stepwise procedure resulted in the preliminary version of the screening tool, i.e., pre-DADYS-Screen.

##### Empirical Investigation of Pre-DADYS-Screen to Develop DADYS-Screen

The quantitative investigation of pre-DADYS-Screen was conducted based on Sample 3, i.e., that is., the community DADYS-Screen development sample (including data of the first participants of ADOPT Epidemiology; *n* = 771) using methods of CTT and IRT. Based on CTT, we investigated item difficulties and item intercorrelations. Furthermore, we determined the number of factors for the model including all items of the pre-DADYS-Screen by parallel analysis (using principal axis analysis as exploratory factor analysis) and we determined factor loadings, for the resulting factor model as well as corrected item-total-correlations, and Cronbach’s alpha. Moreover, we conducted a confirmatory factor analysis and determined model fit, factor loadings, and residual correlations. Within the IRT, the partial credit model with a discrimination parameter fixed to 1 was implied. Item fit was assessed using the so-called infit value based on the standardized mean squared residuals (MNSQ). In addition, category characteristic curves, item characteristic curves and item information functions were inspected. We investigated whether item threshold parameters were monotonously increasing, and residual correlations were below *r* = .40. Furthermore, a potential item bias was investigated by differential item functioning (DIF) analysis on the basis of ordinal logistic regressions with age, sex, parental education, and assessment mode. Uniform DIF means a constant bias across all trait levels, nonuniform DIF means a dependency of the bias on the trait level.

An item was excluded from pre-DADYS-Screen, if (a) an intercorrelation with another item was very low (*r*_ii_*<* .10; please note, if a low correlation was found between 2 items, only one of the questionable items needed to be excluded), (b) the factor loading from principal axis analysis was low (*a*_ij_ < .30), (c) the corrected item-total correlation was low, *r*_i(t-i)_ < .30, (d) the factor loading from confirmatory factor analysis was low, λ_i_ < .40 according to Nunnally (1978), (e) a residual correlation was too high, *r*_res_ >.25 according to Fliege et al. (2005); in case of a residual correlation above the threshold, only one of the questionable 2 items needed to be excluded, (f) IRT-based item infit was not satisfying, MNSQ >1.30 according to Embretson and Reise (2000), (g) threshold parameters were not monotonously increasing, (h) an IRT-based residual correlation was relatively high (*r* > .40), and if (i) DIF was found, the difference in Nagelkerke’s *R*^2^ > .035 according to Zumbo (1999). In general, we did not exclude items only due to low item difficulties found in our community sample (i.e., Sample 3), as we aimed to develop the screening tool for its use in clinical samples.

#### Evaluation of DADYS-Screen

Sample 4 was used to evaluate the final screening tool based on methods from CTT and IRT (i.e., the community DADYS-Screen evaluation sample with data gathered from April 2018 until August 2019; *n* = 8,988). Based on CTT and IRT, we conducted the same analyses as used in the developmental phase and applied the same thresholds. To further test for model fit in IRT analyses, a factor analysis of the residuals was performed. Good model fit is indicated by residual factors with less than 10 % explained variance (Smith, 2002). Besides, the item-person-map and the proportion of persons with not fitting response patterns were calculated.

IBM SPSS Statistics versions 25 to 27 were used for CTT analyses, Mplus version 8.0 for confirmatory factor analyses, and WINMIRA 1.42 and Winsteps 3.67.0 for IRT analyses.

## Results

### Development of DADYS-Screen

#### Generation of the Initial Item Pool

Clinical and research experts gathered items on AD from the following measures: ARI (7 items; Stringaris et al., 2012), ERC (24 items; Shields & Cicchetti, 1997), the PROMIS Anger Scale (5 items; Irwin et al., 2012), the Global Index of the Conners’ Rating Scale (10 items; Conners et al., 2011; Otto et al., 2018), SDQ (10 items; Deutz et al., 2018), and the Disruptive Mood Dysregulation and Irritability Scale from SCL-ODD (11 items; DISYPS-III; Döpfner & Görtz-Dorten, 2017). The generated initial item pool included 67 items and is presented in the Supplementary Material (Table S1).

#### Qualitative

and Quantitative Investigations of The Initial Item Pool to Develop Pre-DADYS-Screen

Using the Delphi rating, a positive consensus was achieved for 17 items (i.e., at least 90 % of the experts agreed that these items should be included in the screening questionnaire). A negative consensus was reached for 7 items, indicating that these items are rather unsuitable to screen children for AD. For the remaining 43 items, no consensus was achieved. Detailed information on the results of the Delphi rating is depicted in Table S2.

In the focus group among clinical experts, 7 items were identified as relevant to screen children for AD by at least 90% of the experts, whereas 8 items were classified as relevant by only 10% or less of the experts (see Table S2). According to the focus groups with parents, the presented items were mostly well understood, some items were rather difficult to answer (especially double-barrelled items [e.g., Items i7 and i25 in Table S2] or those with very long phrasings [e.g., Item i11]).

Quantitative investigations were conducted on the complete initial pool of 67 items using Samples 1 and 2. Sample 1 (i.e., the clinical pre-DADYS-Screen development sample; *n* = 141) includes data gathered for a number of 47 items from the initial pool. Sample 2 (i.e., the population-based pre-DADYS-Screen development sample; *n* = 1,089) includes data gathered for the remaining 20 items of the initial item pool which had been administered in the population-based German BELLA study (Ravens-Sieberer et al., 2015; i.e., 10 items from the Conners’ Global Index (Lidzba et al., 2013; Otto et al., 2018) and 10 items from the SDQ (Deutz et al., 2018)).

Following our stepwise procedure, 30 items were excluded from the initial item pool in the first step because they did not meet the a priori defined qualitative criteria and/or statistical thresholds. In the second step, 23 items were excluded based on a final agreement of experts; excluded items covered the same content as other items but showed less good analyzing results or had limited content validity (e.g., items that were too strongly related to other mental disorders). Overall, this resulted in a remaining set of 14 items that were included in the pre-DADYS-Screen. Results of the conducted analyses and item-specific information on inclusion or reasons for exclusion from pre-DADYS-Screen are shown in Table S2. The pre-DADYS-Screen was subsequently administered in the community sample (Sample 3).

#### Empirical Investigation of Pre-DADYS-Screen to Develop DADYS-Screen

**Figure 2:**
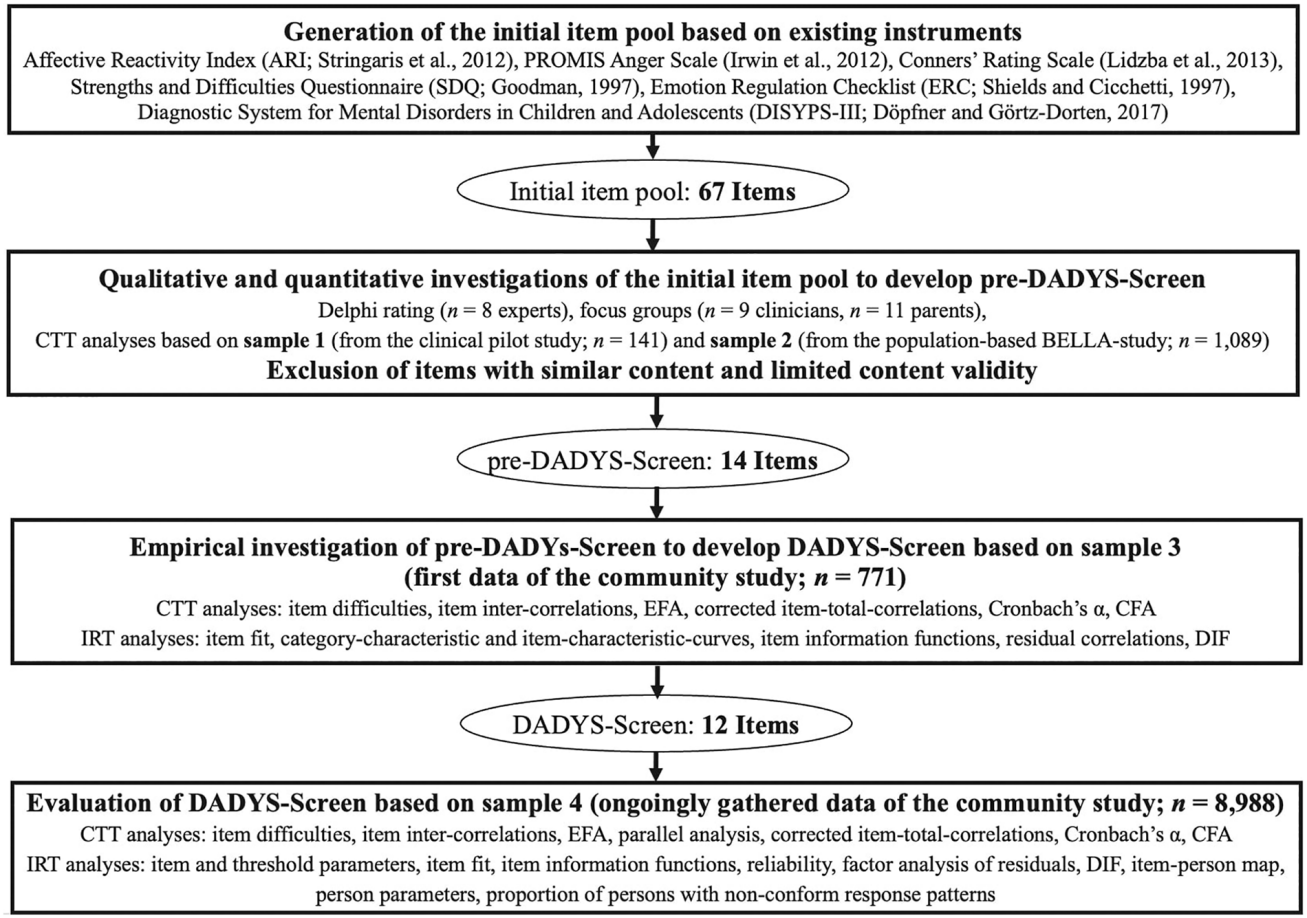
Development and Evaluation Process for the DADYS-Screen *Note.* Samples 1 to 4 are described in more detail in the text (see methods/study and samples). DADYS-Screen = *D*iagnostic Tool for *A*ffective *Dys*regulation in Children—*Screen*ing; CTT = classical test theory; EFA = exploratory factor analysis (principal axis analysis); CFA = confirmatory factor analysis; IRT = item response theory; DIF = differential item functioning.

We investigated pre-DADYS-Screen using data of Sample 3 (i.e., the community DADYS-Screen development sample including data gathered until March 2018; *n* = 771). The results of the parallel analysis indicated a one-factorial solution following Çokluk and Koçak (2016; we used 1,000 simulated data sets; Eigen-values from observed data: first factor = 6.78; second factor = 1.07; Eigen-values from simulated data: first factor = 1.28; second factor = 1.21). Results based on CTT and IRT are provided in Table S3; IRT analyses using alternative software (i.e., the R package ltm) confirmed the results reported based on WINSTEPS. Based on these results, 2 items from the pre-DADYS-Screen were excluded, which resulted in 12 items that were included in DADYS-Screen (presented in Table 2) and subsequently administered in the ongoing community study (Sample 4). Analyses were repeated using only the selected 12 items; additionally, the corresponding model was evaluated by means of a confirmatory factor analysis with mean-and variance-adjusted unweighted least squares (ULSMV)-estimation (χ2 = 315.65, *df* = 54, *p* < .001). We used ULSMV-estimation throughout our analyses with confirmatory factor analysis due to results of statistical studies as gathered by Xia (2016) indicating that ULSMV provides comparable or even slightly better estimates than the more often used mean- and variance-adjusted unweighted least squares estimator. Following the overview of suggested rules of thumb for evaluating model fit as presented by Schermelleh-Engel et al. (2003), the root mean square error of approximation indicated acceptable model fit, RMSEA = 0.078, confidence interval (CI) 90% = [0.070, 0.087], and the comparative fit index (CFI), which evaluates the fit of the proposed model in relation to the more restricted baseline model, reaches the threshold for a good model fit (CFI = 0.974). Figure 2 illustrates the process of developing and evaluating the screening tool.

**Table 2.** Items Included in the Developed DADYS-Screen.

Initial item no.	Final item no.	Item text	Original measure	Response options used in DADYS-Screen
i31	1	Is easily annoyed by others.	ARI	0 = *not at all (true)* 1 = *somewhat (true)* 2 = *mostly (true)* 3 = *especially / very (true)*
i27	2	Is often upset and offended.	DISYPS	
i26	3	Is often irritable or easily annoyed.	DISYPS	
i64	4	Demands must be met immediately—easily frustrated.	CRS	
i14	5	Responds angrily to limit-setting by adults.	ERC	
i20	6	Is impulsive.	ERC	
i32	7	Often loses temper.	ARI	
i8	8	Is prone to angry outbursts/tantrums easily.	ERC	
i35	9	Gets angry frequently.	ARI	
i28	10	Has strong or prolonged temper outbursts with loud scolding, screaming or crying several times a week.	DISYPS	
i33	11	Stays angry for a long time.	ARI	
i2	12	Exhibits wide mood swings (child’s emotional state is difficult to anticipate because she/he moves quickly from positive to negative moods).	ERC	

*Note.* DADYS-Screen = *D*iagnostic Tool for *A*ffective *Dys*regulation in Children—*Screen*ing; ARI = Affective Reactivity Index (Stringaris et al., 2012); DISYPS = Diagnostic System for Mental Disorders in Children and Adolescents (Döpfner & Görtz-Dorten, 2017); CRS = Conners’s Rating Scale (Lidzba et al., 2013); ERC = Emotion Regulation Checklist (Shields & Cicchetti, 1997).

### Evaluation of DADYS-Screen

#### Results Based on Classical Test Theory

Sample 4 was used to evaluate DADYS-Screen (i.e., the community DADYS-Screen evaluation sample including data gathered from April 2018 until August 2019; *n* = 8,988). All participants included in Sample 4 responded to at least 1 item of the DADYS-Screen (*n* = 8,988). Among these 3% had one missing value (*n* = 288) and less than 1% had two missing values (*n* = 39), three missing values (*n* = 14), or four up to 11 missing values (*n* = 14) in the 12 items of the screening tool (per item, missing value rates were consistently below 1 %). For further analyses, we replaced missing values using the Expectation-Maximization (EM) algorithm only if up to three missing values in the items of the screening tool were given; this resulted in Sample 4 under analyses with *n* = 8,974.

Results are presented in Table 3 and gathered in the following. Item difficulties in our community-based sample (i.e., Sample 4) ranged from .08 to .36 across the 12 items (only presented for descriptive purposes). Correlations indicated moderate to strong associations among items (*r_ii_* = .34 to *r_ii_* = .73). The parallel analysis indicated a one-factorial solution (1,000 simulated data sets; Eigen-values from observed data: first factor = 7.19; second factor = 0.93; Eigen-values from simulated data: first factor = 1.07; second factor = 1.05). This one-factor solution explained 60 % of the overall variance among the items with high factor loadings found (from .64 to .86). Corrected item-total correlations were good as well (ranging from .62 to .82). The internal consistency of the 12-item screening tool score was excellent (Cronbach’s α = .94). Furthermore, model fit was determined based on a one-factorial confirmatory factor analysis using ULSMV-estimation (χ^2^ = 4496.21, *df* = 54, *p* < .001), with results indicating “mediocre” fit according to the RMSEA, 0.096, CI (90%) = 0.093–0.098, following Browne and Cudeck (1992), and good fit in relation to the baseline model according to the CFI (0.974) following Schermelleh-Engel et al. (2003). Factor loadings from confirmatory factor analysis ranged from .70 to .92 across the items and residual correlations were consistently below the threshold (between −.06 and .19). Sensitivity analyses revealed that these results hold for the subsample of those with complete data in all items of the screening tool (*n* = 8,633).

**Table 3.** Item-Specific Results Based on Classical Test Theory and Confirmatory Factor Analysis for the Evaluation of the DADYS-Screen.

Final item no.	Item difficulty (*P*_i_)	Item-intercorrelations (*r*_ii_)	Factor loading from PAA (*a*_ij_)	Corrected item-total correlation (*r*_i[t-i]_)	Internal consistency, if item deleted (α_i_)	Factor loading from CFA (λ_i_)	Residual correlations (*r*_res_)
1	.36	.34–.67	0.64	.63	.94	.70	−.05 to .19
2	.30	.42–.73	0.76	.74	.93	.82	−.05 to .15
3	.30	.43–.73	0.80	.79	.93	.87	−.05 to .19
4	.23	.37–.57	0.67	.65	.94	.73	−.05 to .15
5	.33	.39–.57	0.70	.68	.93	.76	−.05 to .15
6	.28	.39–.63	0.73	.70	.93	.78	−.05 to .08
7	.18	.47–.70	0.84	.81	.93	.91	−.05 to .08
8	.20	.45–.71	0.85	.81	.93	.90	−.05 to .05
9	.20	.48–.71	0.86	.82	.93	.92	−.03 to .05
10	.10	.38–.61	0.76	.73	.93	.90	−.06 to .07
11	.08	.34–.56	0.65	.62	.94	.78	−.05 to .07
12	.15	.39–.53	0.71	.69	.93	.79	−.03 to .06

*Note. n* = 8,974 children aged 8 to 12 years from the evaluation sample with less than 3 items missing, up to three missing values were replaced by the Expectation-Maximization (EM) algorithm. DADYS-Screen = *D*iagnostic Tool for *A*ffective *Dys*regulation in Children—*Screen*ing; PAA = principal axis analysis; CFA = confirmatory factor analysis.

#### Results Based on Item Response Theory

**Figure 3.**
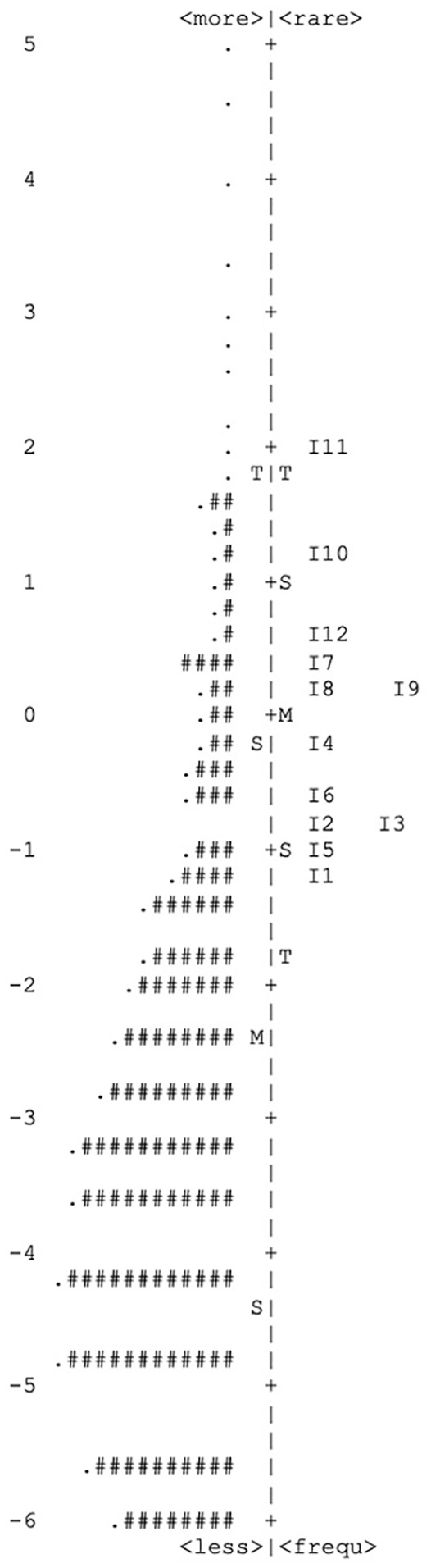
Item-Person Map: Location of Persons and Items on the Latent Trait Affective Dysregulation in a Community Sample of n = 8,974 Children Aged 8 to 12 Years *Note.* axis = latent trait score, negative values indicate lower affective dysregulation and vice versa; S = 1 standard deviation unit; T = 2 standard deviation units; each # represents 61 persons; I = item with final item number.

**Figure 4.**
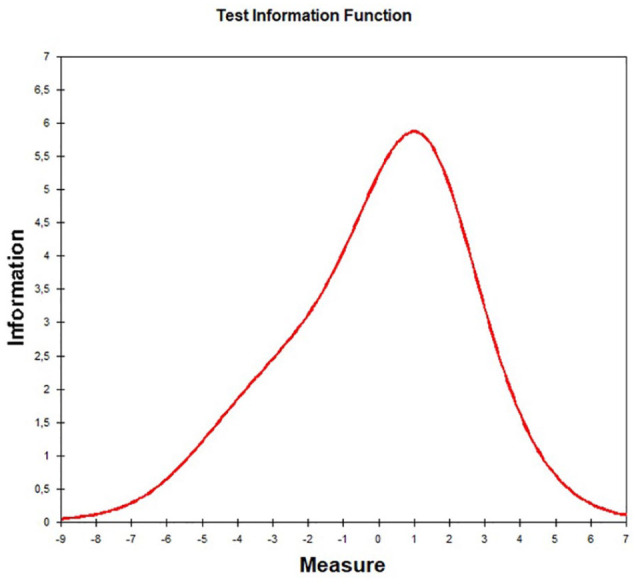
Test Information Function *Note.* Sum of the item information function. x-axis = latent trait score; y-axis = amount of information obtained by all items.

Table 4 shows the item statistics according to the probabilistic test theory following the partial credit model generated for Sample 4 under analyses (*n* = 8,974). The item parameters (equivalent to item difficulty in the CTT, i.e., location of the items on the latent trait) ranged from −1.29 to 1.92. The threshold parameters (location of category boundaries) were monotonically increasing for all items and ranged from −5.00 to 3.69. The item fit was good for all items. The item information functions were accordingly bell-shaped with the exception of Item i14 (Final Item No. 5: “Responds angrily to limit-setting by adults”), i31 (Final Item No. 1: “Is easily annoyed by others”), and i27 (Final Item No. 2: “Is often upset and offended”) with a slight bimodality. The Rasch reliability was good (i.e., *R* = .88). A factor analysis of the residuals should not yield residual factors with more than 10% explained variance as desired. In fact, the explained variance of the first contrast was only 6.5%. The analysis on DIF using ordinal logistic regression showed no effects in the sense of an item bias by age, sex, parental education, or assessment mode (all values < .035). The item-person map in Figure 3 shows the correspondence of persons and items along with the latent trait. The person parameters of the present sample are mainly in a very low (negative) range of values. The item locations are relatively close around the mean and relatively close to each other. The person parameters were normally distributed, but truncated to the left, and correlated strongly with the raw score values (*r* = .95). The proportion of participants with response patterns that violate the model was low (3.7%). Figure 4 shows the resulting test information function, which is close to the desired normal curve, but more leptokurtic.

**Table 4.** Item Statistics According to the Probabilistic Test Theory (Partial Credit Model) for the Evaluation of the DADYS-Screen.

Final item no.	Item location	Threshold locations			Item fit (MNSQ)	DIF							
						Age		Sex		Parental education		Paper-pencil vs. online assessment	
		0/1	1/2	2/3		Uniform	Nonuniform	Uniform	Nonuniform	Uniform	Nonuniform	Uniform	Nonuniform
1	−1.29	−5.00	−0.47	1.58	1.25	.001	.001	.000	.001	.000	.000	.000	.000
2	−0.75	−3.78	−0.24	1.77	0.93	.000	.000	.000	.000	.001	.001	.000	.000
3	−0.80	−3.53	−0.44	1.57	0.80	.000	.001	.000	.000	.002	.002	.000	.000
4	−0.97	−4.31	−0.14	1.53	1.30	.000	.000	.000	.000	.000	.000	.000	.000
5	−0.64	−3.17	−0.30	1.55	1.13	.001	.001	.001	.001	.001	.001	.001	.001
6	0.15	−1.97	0.47	1.94	1.13	.000	.000	.000	.000	.001	.001	.000	.001
7	0.20	−2.23	0.55	2.27	0.72	.000	.001	.002	.002	.002	.002	.001	.001
8	1.23	0.01	1.31	2.36	0.75	.002	.002	.001	.001	.000	.000	.000	.000
9	1.92	−0.15	2.21	3.69	0.69	.000	.000	.000	.000	.000	.000	.000	.000
10	0.66	−1.20	0.96	2.21	0.84	.002	.002	.000	.000	.000	.000	.000	.000
11	−1.29	−5.00	−0.47	1.58	1.14	.001	.001	.000	.001	.001	.001	.001	.001
12	−0.75	−3.78	−0.24	1.77	1.17	.004	.004	.008	.008	.001	.001	.000	.000

*Note. n* = 8,974 children aged 8 to 12 years; Nagelkerke’s *R*^2^; DADYS-Screen = *D*iagnostic Tool for *A*ffective *Dys*regulation in Children—*Screen*ing; MNSQ = mean squared residuals; DIF = differential item functioning.

## Discussion

The present study describes the development and evaluation of a screening questionnaire based on parent reports to assess AD in 8- to 12-year-old children. We used qualitative methods like Delphi rating of experts and focus groups with clinicians and parents, and quantitative methods based on CTT and IRT investigating clinical and population-based data. The resulting 12 items of the DADYS-Screen are appropriate and suitable for assessing AD comprehensively due to ratings of clinical experts and comprehensible according to the feedback of parents. The evaluation of the DADYS-Screen demonstrated good feasibility and mainly good psychometric properties according to parameters based on CTT and IRT.

We followed the state of the art in our proceeding using a mixed-methods approach with recommended methods. Furthermore, we chose the items for the initial item pool from existing well-established and validated instruments, such as the Affective Reactivity Index (Stringaris et al., 2012) and the Emotion Regulation Checklist (Shields & Cicchetti, 1997). Moreover, we followed Boateng et al. (2018) and used a deductive method in developing our screening tool. Our item pool covered a broad and comprehensive construct, which was somewhat broader than the target construct of AD and included 5 times as many items as gathered in the final DADYS-Screen (Boateng et al., 2018; Weiner et al., 2012).

In our evaluation of the screening measure, we detected overall good psychometric properties according to the CTT. We found good item-intercorrelations, high factor loadings, good internal consistency, and low residual correlations. Model fit according to confirmatory factor analysis was good in comparison to the baseline model, although the fit was only “mediocre” according to guidelines for interpreting the RMSEA of Brown and Cudeck (1992). We tolerated low item difficulties as our data was gathered in a community sample, but the main target group for the screening tool will be children under risk of AD and clinical samples. The analyses in the probabilistic model on the evaluation of DADYS-Screen confirmed the good measuring characteristics of the scale overall. Although the item difficulties were located rather closely, the category difficulties were sufficiently broadly spread. The slight bimodality of the information functions of Items i14, i27, and i31 (i.e., final item numbers 5, 2 and 1) should be further investigated in future studies. All other characteristics showed a good fit of the Rasch model to the data, also an item bias could not be identified. The item-person map (Figure 3) indicates that the items are somewhat too difficult for a community sample because the location of the item parameters differs somewhat from the location of the person parameters (and reliability is best when both correspond to each other). However, as the target group of the instrument are selected subjects with an elevated symptom level of AD, the screening tool will probably show a better fit in corresponding clinical samples; this will be investigated in future studies.

The parallel analysis indicated a one-factorial solution. However, two-factor models for tonic (irritable mood) and phasic (temper outbursts) irritability are also described in the literature (Klein et al., 2021; Silver et al., 2021). We will consider this in future analyses based on clinical data. We will validate our screening tool based on clinical data collected in the ADOPT consortium. Analyses based on future clinical data will include the determination of item difficulties and the investigation of the factor structure of the screening tool in the clinical population, and we aim to provide a cutoff for our screening measure considering its sensitivity and specificity due to relevant psychiatric diagnoses.

The construct of AD is very closely related to the new diagnosis of DMDD. According to the *DSM-5*, DMDD is set into the section of mood disorders, but in the ICD-11, AD will be a subcategory of oppositional defiant disorder. It thus seems that AD can be seen as a phenomenon that occurs in both internalizing and externalizing disorders. Research has shown that internalizing problems of children are often underestimated by parents (Dolle et al 2012), but externalizing problems are more obvious and better observable by parents (Holmbeck et al., 2002; Klasen et al., 2016). Our screening tool on AD gathers parent reports. For further research on AD (including studies comparing parent- and self-reports) and clinical practice, a screening measure for gathering self-reports on AD in children is needed as well. Thus, a self-reported version of our screening tool will be provided by the ADOPT consortium. Both measures could be useful and easily administered in studies and clinical practice investigating AD as a potential transdiagnostic dimension.

Our study has some limitations. First, many different terms are used in the literature to characterize very similar constructs, and a clear consensus definition of what constitutes AD or irritability remains elusive. In line with the literature (Döpfner et al., 2019; Giller et al., 2021; Waltereit et al., 2019), we chose the term AD, which appears to be less established but focuses on a more narrow construct of excessive reactivity to negative emotional stimuli with affective and behavioral components, differentiating it from broader concepts such as emotion dysregulation. Second, considering the developmental context of prepuberty (e.g., hormonal development), our study focused on children aged 8 to 12 years. We did not develop our screening tool for children younger than 8 years or older than 12 years. Future research may wish to investigate the feasibility and validity of our screening tool for use in younger or older children. Third, the validation of the DADYS-Screen and its self-reported version is not provided so far but will add important information for their use, especially in samples under risk of AD. The present study does not provide information on the construct and criterion validity of DADYS-Screen. The questionnaire used for the screening of participants at risk of AD in the community study was kept as short as possible to support the participation of as many willing parents as possible. However, in analyses conducted for another article based on data of Sample 4, a strong correlation was found between the PROMIS Anger Scale (including five items measuring parent-reported irritable and angry mood of the child) and the DADYS-Screen (*r* = .78; *p*< .001; Kaman et al., 2021). The planned validation of the DADYS-Screen will include further investigations besides the determination of a cutoff based on data gathered in further subprojects of the ongoing overall ADOPT study. Our study has some strengths as well. We included items from well-established measures, followed a mixed-methods approach and analyzed a large community-based sample. Furthermore, especially in the development of the screening tool, a strong team of clinical and research experts cooperated very closely considering additional focus groups results gathered in parents.

Overall, this study provides preliminary evidence for the psychometric performance of the DADYS-Screen and supports its use in identifying children with persistent irritability and severe temper outbursts, although further psychometric investigation using clinical data is needed. DADYS-Screen seems to allow clinicians and researchers a better identification of children with AD and is thus promising to support appropriate diagnosis, treatment, and clinical practice.

## Supplemental Material

sj-docx-1-asm-10.1177_10731911221082709 – Supplemental material for The DADYS-Screen: Development and Evaluation of a Screening Tool for Affective Dysregulation in Children Click here for additional data file. Supplemental material, sj-docx-1-asm-10.1177_10731911221082709 for The DADYS-Screen: Development and Evaluation of a Screening Tool for Affective Dysregulation in Children by Christiane Otto, Anne Kaman, Claus Barkmann, Manfred Döpfner, Anja Görtz-Dorten, Claudia Ginsberg, Sara Zaplana Labarga, Anne-Katrin Treier, Veit Roessner, Charlotte Hanisch, Michael Koelch, Tobias Banaschewski and Ulrike Ravens-Sieberer in Assessment
