# Supplementary material for: The DADYS-Screen: Development and Evaluation of a Screening Tool for Affective Dysregulation in Children
Source: Assessment. 2022 Mar 18;30(4):1080–94. doi: 10.1177/10731911221082709 (PMC10152573; doi:10.1177/10731911221082709)
Supplement: sj-docx-1-asm-10.1177_10731911221082709 – Supplemental material for The DADYS-Screen: Development and Evaluation of a Screening Tool for Affective Dysregulation in Children [file sj-docx-1-asm-10.1177_10731911221082709.docx]

**Supplementary Material**

**Manuscript title:** The DADYS-Screen – Development and Evaluation of a Screening Tool for Affective Dysregulation in Children

**Journal:** Assessment

*Table S1.*

*Items included in the item pool*

| Initial item no. | Item text | Instrument of origin |
| --- | --- | --- |
| i1 | Is a cheerful child. | ERC |
| i2 | Exhibits wide mood swings (child’s emotional state is difficult to anticipate because s/he moves quickly from positive to negative moods). | ERC |
| i3 | Responds positively to neutral or friendly approaches by adults. | ERC |
| i4 | Transitions well from one activity to another; does not become anxious, angry, distressed or overly excited when moving from one activity to another. | ERC |
| i5 | Can recover quickly from episodes of upset or distress (e.g. does not pout or remain sullen, anxious or sad after emotionally distressing events). | ERC |
| i6 | Is easily frustrated. | ERC |
| i7 | Responds positively to neutral or friendly approaches by peers. | ERC |
| i8 | Is prone to angry outbursts / tantrums easily. | ERC |
| i9 | Is able to delay gratification (wait for good things). | ERC |
| i10 | Takes pleasure in the distress of others (e.g. laughs when another person gets hurt or punished; enjoy teasing others). | ERC |
| i11 | Can modulate excitement in emotionally arousing situations (e.g. does not get ‘carried away’ in high-energy situations, or overly excited in inappropriate contexts. | ERC |
| i12 | Is whiny or clingy with adults. | ERC |
| i13 | Is prone to disruptive outbursts of energy and exuberance. | ERC |
| i14 | Responds angrily to limit-setting by adults. | ERC |
| i15 | Can say when s/he is feeling sad, angry or mad, fearful or afraid. | ERC |
| i16 | Seems sad or listless. | ERC |
| i17 | Is overly exuberant when attempting to engage other in play. | ERC |
| i18 | Displays flat affect (expression is vacant and inexpressive; child seems emotionally absent). | ERC |
| i19 | Responds negatively to neutral or friendly approaches by peers (e.g. may speak in an angry tone of voice or respond fearfully). | ERC |
| i20 | Is impulsive. | ERC |
| i21 | Is empathic towards others; shows concern when others are upset or distressed. | ERC |
| i22 | Displays exuberance that others find intrusive or disruptive. | ERC |
| i23 | Displays appropriate negative emotions (anger, fear, frustration, distress) in response to hostile, aggressive or intrusive acts by peers. | ERC |
| i24 | Displays negative emotions when attempting to engage others in play. | ERC |
| i25 | Gets angry quickly or has unusually frequent or severe temper outbursts for his age. | DISYPS |
| i26 | Is often irritable or easily annoyed. | DISYPS |
| i27 | Is often upset and offended. | DISYPS |
| i28 | Has strong or prolonged temper outbursts with loud scolding, screaming or crying several times a week. | DISYPS |
| i29 | Has strong or prolonged temper outbursts several times a week during which he/she becomes physically aggressive or destroys objects. | DISYPS |
| i30 | Is in a bad or irritable mood most of the time. | DISYPS |
| i31 | Is easily annoyed by others. | ARI |
| i32 | Often loses temper. | ARI |
| i33 | Stays angry for a long time. | ARI |
| i34 | Is angry most of the time. | ARI |
| i35 | Gets angry frequently. | ARI |
| i36 | Loses temper easily. | ARI |
| i37 | Overall, irritability causes him/her problems. | ARI |
| i38 | My child felt mad. | PROMIS Anger |
| i39 | My child was so angry he/she felt like yelling at somebody. | PROMIS Anger |
| i40 | My child was so angry he/she felt like throwing something. | PROMIS Anger |
| i41 | My child felt upset. | PROMIS Anger |
| i42 | When my child got mad, he/she stayed mad. | PROMIS Anger |
| i43 | The problems mentioned have a significant impact on relationships with other family members (e.g. parents, siblings). | DISYPS |
| i44 | The problems mentioned have a significant impact on relationships with adults not being part of the family (e.g. teachers). | DISYPS |
| i45 | The problems mentioned have a significant impact on relationships with other children and adolescents and on the participation in leisure activities. | DISYPS |
| i46 | The problems mentioned have a significant impact on school performance. | DISYPS |
| i47 | She/he suffers considerably from the problems mentioned. | DISYPS |
| i48 | Restless, overactive, cannot stay still for long. | SDQ |
| i49 | Often loses temper. | SDQ |
| i50 | Generally well behaved, usually does what adults request. | SDQ |
| i51 | Constantly fidgeting or squirming. | SDQ |
| i52 | Often fights with other youth or bullies them. | SDQ |
| i53 | Easily distracted, concentration wanders. | SDQ |
| i54 | Often lies or cheats. | SDQ |
| i55 | Thinks things out before acting. | SDQ |
| i56 | Steals from home, school or elsewhere. | SDQ |
| i57 | Good attention span, sees work through to the end. | SDQ |
| i58 | Restless or overactive. | CRS |
| i59 | Excitable, impulsive. | CRS |
| i60 | Disturbs other children. | CRS |
| i61 | Fails to finish things he/she starts - short attention span. | CRS |
| i62 | Constantly fidgeting. | CRS |
| i63 | Easily distracted. | CRS |
| i64 | Demands must be met immediately - easily frustrated. | CRS |
| i65 | Cries often and easily. | CRS |
| i66 | Mood changes quickly and drastically. | CRS |
| i67 | Temper outbursts, explosive or unpredictable behaviour. | CRS |

*Note.* ERC = Emotion Regulation Checklist (Shields & Cicchetti, 1997); DISYPS = Diagnostic System for Mental Disorders in Children and Adolescents (Döpfner & Görtz-Dorten, 2017); ARI = Affective Reactivity Index (Stringaris et al., 2012); PROMIS Anger = PROMIS Parent Proxy Anger Scale (Pilkonis et al., 2011); SDQ = Strengths and Difficulties Questionnaire (Goodman, 1997); CRS = Conners’ Rating Scale (Lidzba et al., 2013)

*Table S2.*

*Development of pre-DADYS-Screen based on results of qualitative and quantitative methods (using clinical and population-based data)*

| Initial item no. |  | Qualitative analyses | | |  | Quantitative analyses based on classical test theory | | | | | | |  | Decision based on stepwise procedure and final agreement among experts |
| --- | --- | --- | --- | --- | --- | --- | --- | --- | --- | --- | --- | --- | --- | --- |
|  |  | Delphi rating  (% of experts in consensus for inclusion) | Clinician FG (% who rated the item as relevant) | Parent FG  (comprehen-sibility of item) |  | Clinical sample | | |  | Population-based sample | | |  |  |
|  |  |  |  |  |  | Item difficulty  (*P*_i_) | Factor loading  (*a*_ij_) | Corrected Item-total correlation (*r*_i(t-i)_) |  | Item difficulty  (*P*_i_) | Factor loading  (*a*_ij_) | Corrected Item-total correlation (*r*_i(t-i)_) |  |  |
| i1 |  | 67% | 22% | comprehensible |  | 0.64 | **0.24** | **0.25** |  |  |  |  |  | excluded (qualitative criteria and/or statistical thresholds not met) |
| i2 |  | 100% | 56% | comprehensible |  | 0.42 | 0.69 | 0.68 |  |  |  |  |  | **pre-DADYS-Screen** |
| i3 |  | 33% | 22% | **difficult to comprehend** |  | 0.76 | **0.26** | **0.28** |  |  |  |  |  | excluded (qualitative criteria and/or statistical thresholds not met) |
| i4 |  | 17% | 11% | mostly comprehensible |  | 0.57 | 0.35 | 0.36 |  |  |  |  |  | excluded (limited content validity) |
| i5 |  | 83% | 78% | comprehensible |  | 0.51 | 0.44 | 0.45 |  |  |  |  |  | excluded (content very similar to i33, which performed better) |
| i6 |  | 100% | 100% | comprehensible |  | 0.54 | 0.60 | 0.59 |  |  |  |  |  | excluded (limited content validity) |
| i7 |  | 33% | 22% | **difficult to comprehend** |  | 0.78 | **0.25** | **0.27** |  |  |  |  |  | excluded (qualitative criteria and/or statistical thresholds not met) |
| i8 |  | 100% | 89% | comprehensible |  | 0.43 | 0.70 | 0.68 |  |  |  |  |  | **pre-DADYS-Screen** |
| i9 |  | 50% | 44% | comprehensible |  | 0.50 | 0.46 | 0.45 |  |  |  |  |  | **pre-DADYS-Screen** |
| i10 |  | 33% | 22% | comprehensible |  | **0.15** | 0.47 | 0.48 |  |  |  |  |  | excluded (qualitative criteria and/or statistical thresholds not met) |
| i11 |  | 67% | 11% | **difficult to comprehend** |  | 0.45 | 0.54 | 0.54 |  |  |  |  |  | excluded (qualitative criteria and/or statistical thresholds not met) |
| i12 |  | 50% | **0%** | comprehensible |  | **0.19** | 0.30 | 0.32 |  |  |  |  |  | excluded (qualitative criteria and/or statistical thresholds not met) |
| i13 |  | 100% | 56% | **difficult to comprehend** |  | 0.35 | 0.56 | 0.56 |  |  |  |  |  | excluded (qualitative criteria and/or statistical thresholds not met) |
| i14 |  | 83% | 89% | comprehensible |  | 0.49 | 0.70 | 0.69 |  |  |  |  |  | **pre-DADYS-Screen** |
| i15 |  | 17% | 44% | comprehensible |  | 0.60 | 0.32 | 0.34 |  |  |  |  |  | excluded (limited content validity) |
| i16 |  | 67% | 33% | mostly comprehensible |  | 0.29 | **0.24** | **0.25** |  |  |  |  |  | excluded (qualitative criteria and/or statistical thresholds not met) |
| i17 |  | 33% | 22% | comprehensible |  | 0.28 | 0.40 | 0.41 |  |  |  |  |  | excluded (limited content validity) |
| i18 |  | 17% | 22% | comprehensible |  | **0.14** | **0.14** | **0.16** |  |  |  |  |  | excluded (qualitative criteria and/or statistical thresholds not met) |
| i19 |  | 50% | 89% | comprehensible |  | **0.08** | 0.37 | 0.39 |  |  |  |  |  | excluded (qualitative criteria and/or statistical thresholds not met) |
| i20 |  | 83% | 78% | comprehensible |  | 0.50 | 0.74 | 0.71 |  |  |  |  |  | **pre-DADYS-Screen** |
| i21 |  | 17% | 33% | comprehensible |  | 0.63 | 0.42 | 0.44 |  |  |  |  |  | **pre-DADYS-Screen** |
| i22 |  | 17% | 11% | comprehensible |  | 0.28 | 0.53 | 0.52 |  |  |  |  |  | excluded (limited content validity) |
| i23 |  | 80% | 22% | mostly comprehensible |  | 0.57 | **0.01** | **0.02** |  |  |  |  |  | excluded (qualitative criteria and/or statistical thresholds not met) |
| i24 |  | 50% | 67% | comprehensible |  | **0.17** | 0.39 | 0.41 |  |  |  |  |  | excluded (qualitative criteria and/or statistical thresholds not met) |
| i25 |  | 100% | 89% | **difficult to comprehend** |  | 0.36 | 0.81 | 0.77 |  |  |  |  |  | excluded (qualitative criteria and/or statistical thresholds not met) |
| i26 |  | 100% | 100% | comprehensible |  | 0.50 | 0.82 | 0.79 |  |  |  |  |  | **pre-DADYS-Screen** |
| i27 |  | 100% | 89% | comprehensible |  | 0.38 | 0.76 | 0.74 |  |  |  |  |  | **pre-DADYS-Screen** |
| i28 |  | 100% | 100% | comprehensible |  | 0.26 | 0.73 | 0.71 |  |  |  |  |  | **pre-DADYS-Screen** |
| i29 |  | 100% | 100% | comprehensible |  | **0.14** | 0.62 | 0.60 |  |  |  |  |  | excluded (qualitative criteria and/or statistical thresholds not met) |
| i30 |  | 83% | 100% | comprehensible |  | **0.17** | 0.63 | 0.64 |  |  |  |  |  | excluded (qualitative criteria and/or statistical thresholds not met) |
| i31 |  | 83% | 89% | comprehensible |  | 0.50 | 0.71 | 0.69 |  |  |  |  |  | **pre-DADYS-Screen** |
| i32 |  | 100% | 100% | comprehensible |  | 0.38 | 0.85 | 0.82 |  |  |  |  |  | **pre-DADYS-Screen** |
| i33 |  | 67% | 78% | mostly comprehensible |  | 0.23 | 0.69 | 0.67 |  |  |  |  |  | **pre-DADYS-Screen** |
| i34 |  | 67% | 67% | mostly comprehensible |  | **0.15** | 0.69 | 0.67 |  |  |  |  |  | excluded (qualitative criteria and/or statistical thresholds not met) |
| i35 |  | 100% | 89% | comprehensible |  | 0.31 | 0.81 | 0.77 |  |  |  |  |  | **pre-DADYS-Screen** |
| i36 |  | 100% | 89% | comprehensible |  | 0.36 | 0.84 | 0.81 |  |  |  |  |  | excluded (limited content validity) |
| i37 |  | 83% | 56% | comprehensible |  | 0.35 | 0.77 | 0.75 |  |  |  |  |  | excluded (limited content validity, captures impairment rather than symptoms) |
| i38 |  | **0%** | 13% | **difficult to comprehend** |  | 0.42 | 0.73 | 0.70 |  |  |  |  |  | excluded (qualitative criteria and/or statistical thresholds not met) |
| i39 |  | **0%** | 33% | **difficult to comprehend** |  | 0.36 | 0.76 | 0.73 |  |  |  |  |  | excluded (qualitative criteria and/or statistical thresholds not met) |
| i40 |  | **0%** | 33% | **difficult to comprehend** |  | 0.26 | 0.70 | 0.67 |  |  |  |  |  | excluded (qualitative criteria and/or statistical thresholds not met) |
| i41 |  | 17% | 33% | **difficult to comprehend** |  | 0.49 | 0.74 | 0.71 |  |  |  |  |  | excluded (qualitative criteria and/or statistical thresholds not met) |
| i42 |  | 50% | 56% | comprehensible |  | 0.24 | 0.69 | 0.68 |  |  |  |  |  | excluded (content very similar to i33, which performed better) |
| i43 |  | 83% | 56% | comprehensible |  | 0.37 | 0.67 | 0.66 |  |  |  |  |  | excluded (limited content validity, captures impairment rather than symptoms) |
| i44 |  | 83% | 56% | comprehensible |  | 0.30 | 0.67 | 0.67 |  |  |  |  |  | excluded (limited content validity, captures impairment rather than symptoms) |
| i45 |  | 83% | 56% | mostly comprehensible |  | 0.33 | 0.64 | 0.64 |  |  |  |  |  | excluded (limited content validity, captures impairment rather than symptoms) |
| i46 |  | 83% | 44% | comprehensible |  | 0.32 | 0.66 | 0.65 |  |  |  |  |  | excluded (limited content validity, captures impairment rather than symptoms) |
| i47 |  |  |  | comprehensible |  | 0.40 | 0.71 | 0.70 |  |  |  |  |  | excluded (limited content validity, captures impairment rather than symptoms) |
| i48 |  | **0%** | **0%** | comprehensible |  |  |  |  |  | 0.28 | 0.65 | 0.60 |  | excluded (qualitative criteria and/or statistical thresholds not met) |
| i49 |  | 100% | 89% | comprehensible |  |  |  |  |  | 0.28 | 0.53 | 0.51 |  | excluded (content very similar to i35, which performed better) |
| i50 |  | 17% | 11% | mostly comprehensible |  |  |  |  |  | 0.71 | 0.39 | 0.38 |  | excluded (limited content validity) |
| i51 |  | 17% | **0%** | comprehensible |  |  |  |  |  | 0.19 | 0.68 | 0.63 |  | excluded (qualitative criteria and/or statistical thresholds not met) |
| i52 |  | 67% | 33% | mostly comprehensible |  |  |  |  |  | 0.14 | 0.41 | 0.39 |  | excluded (limited content validity) |
| i53 |  | 17% | 11% | comprehensible |  |  |  |  |  | 0.45 | 0.66 | 0.61 |  | excluded (limited content validity) |
| i54 |  | 17% | 25% | comprehensible |  |  |  |  |  | 0.20 | 0.42 | 0.40 |  | excluded (limited content validity) |
| i55 |  | 17% | 33% | **difficult to comprehend** |  |  |  |  |  | 0.65 | 0.51 | 0.48 |  | excluded (qualitative criteria and/or statistical thresholds not met) |
| i56 |  | 17% | 22% | mostly comprehensible |  |  |  |  |  | 0.02 | **0.13** | **0.13** |  | excluded (qualitative criteria and/or statistical thresholds not met) |
| i57 |  | **0%** | **0%** | mostly comprehensible |  |  |  |  |  | 0.63 | 0.62 | 0.58 |  | excluded (qualitative criteria and/or statistical thresholds not met) |
| i58 |  | **0%** | **0%** | comprehensible |  |  |  |  |  | 0.26 | 0.65 | 0.59 |  | excluded (qualitative criteria and/or statistical thresholds not met) |
| i59 |  | 100% | 67% | mostly comprehensible |  |  |  |  |  | 0.32 | 0.63 | 0.61 |  | excluded (content very similar to i20, which performed better) |
| i60 |  | 50% | 11% | comprehensible |  |  |  |  |  | 0.13 | 0.61 | 0.58 |  | excluded (limited content validity) |
| i61 |  | **0%** | **0%** | comprehensible |  |  |  |  |  | 0.22 | 0.61 | 0.57 |  | excluded (qualitative criteria and/or statistical thresholds not met) |
| i62 |  | 17% | **0%** | comprehensible |  |  |  |  |  | 0.20 | 0.75 | 0.69 |  | excluded (qualitative criteria and/or statistical thresholds not met) |
| i63 |  | 17% | **0%** | comprehensible |  |  |  |  |  | 0.32 | 0.70 | 0.66 |  | excluded (qualitative criteria and/or statistical thresholds not met) |
| i64 |  | 83% | 44% | mostly comprehensible |  |  |  |  |  | 0.28 | 0.49 | 0.47 |  | **pre-DADYS-Screen** |
| i65 |  | 100% | 56% | comprehensible |  |  |  |  |  | 0.17 | **0.29** | **0.28** |  | excluded (qualitative criteria and/or statistical thresholds not met) |
| i66 |  | 100% | 89% | mostly comprehensible |  |  |  |  |  | 0.20 | 0.47 | 0.46 |  | excluded (content very close to i2, which performed better) |
| i67 |  | 100% | 100% | comprehensible |  |  |  |  |  | 0.17 | 0.57 | 0.56 |  | excluded (content very close to i28, which performed better) |

*Note.* Results are printed in bold, if not matching a priori defined criteria with not considering item difficulties determined based on the population-based sample (see manuscript text, Methods/Analyses); positively phrased items were recoded prior to exploratory factor analysis and calculating corrected item-total-correlations; FG=focus group.

*Table S3.*

*Development of DADYS-Screen based on results of classical test theory, confirmatory factor analysis and item response theory (n = 771)*

| Initial item no. | Results of CTT analyses | | | | | |  | | Results of CFA** | | |  | | Results of IRT analyses | | | | | |  | Decision based on statistical results | |  |  |
| --- | --- | --- | --- | --- | --- | --- | --- | --- | --- | --- | --- | --- | --- | --- | --- | --- | --- | --- | --- | --- | --- | --- | --- | --- |
|  | Item difficulty  (*P*_i_) | Item-inter-correlations  (*r*_ii_) | Factor loading  (*a*_ij_) | Corrected item-total correlation | Cronbach’s α if item deleted* |  | | Factor loading  (λ_i_) | | Residual correlations (*r*_res_) |  | | Itemfit  (MSNQ) | | CCC | ICC | IIF | max. residual correlations | DIF max |  | |  | | |
| i2 | 0.14 | .22-.56 | 0.73 | 0.68 | 0.90 |  | | 0.82 | | -.06-.07 |  | | 0.87 | | + | + | + | -.20 | .008 |  | | **DADYS-Screen** | | |
| i8 | 0.22 | .21-.69 | 0.83 | 0.78 | 0.90 |  | | 0.89 | | -.08-.05 |  | | 0.67 | | + | + | + | -.19 | .007 |  | | **DADYS-Screen** | | |
| i9 | 0.61 | .19-.40 | 0.40 | 0.40 | 0.91 |  | | 0.44 | | -.06-.19 |  | | **1.54** | | + | + | + | -.29 | .003 |  | | excluded | | |
| i14 | 0.31 | .19-.47 | 0.58 | 0.57 | 0.90 |  | | 0.64 | | -.05-.15 |  | | 1.05 | | + | + | + | -.16 | .003 |  | | **DADYS-Screen** | | |
| i20 | 0.33 | **.07 with i21**  .27-.55 | 0.61 | 0.58 | 0.90 |  | | 0.66 | | -.12-.10 |  | | 1.15 | | + | + | + | -.21 | .002 |  | | **DADYS-Screen** | | |
| i21 | 0.74 | **.07 with i20**  .14-.24 | 0.31 | 0.30 | 0.91 |  | | **0.33** | | -.12-.12 |  | | **1.67** | | + | + | + | -.24 | .011 |  | | excluded | | |
| i26 | 0.26 | .16-.66 | 0.78 | 0.75 | 0.90 |  | | 0.85 | | -.09-.18 |  | | 0.72 | | + | + | + | -.29 | .004 |  | | **DADYS-Screen** | | |
| i27 | 0.21 | .16-.66 | 0.67 | 0.63 | 0.90 |  | | 0.74 | | -.09-.18 |  | | 0.93 | | + | + | + | .32 | .003 |  | | **DADYS-Screen** | | |
| i28 | 0.08 | .14-.57 | 0.72 | 0.67 | 0.90 |  | | 0.89 | | -.06-.08 |  | | 0.79 | | + | + | + | -.19 | .003 |  | **DADYS-Screen** | | | |
| i31 | 0.39 | .18-.60 | 0.60 | 0.57 | 0.90 |  | | 0.65 | | -.06-.18 |  | | 1.10 | | + | + | + | .23 | .004 |  | **DADYS-Screen** | | |  |
| i32 | 0.18 | .23-.69 | 0.84 | 0.80 | 0.90 |  | | 0.91 | | -.06-.10 |  | | 0.63 | | + | + | + | -.26 | .003 |  | **DADYS-Screen** | | | |
| i33 | 0.07 | .16-.48 | 0.58 | 0.54 | 0.91 |  | | 0.73 | | -.06-.08 |  | | 1.01 | | + | + | + | -.13 | .010 |  | **DADYS-Screen** | | | |
| i35 | 0.21 | .24-.69 | 0.85 | 0.81 | 0.90 |  | | 0.91 | | -.04-.05 |  | | 0.61 | | + | + | + | .23 | .005 |  | **DADYS-Screen** | | | |
| i64 | 0.21 | .23-.48 | 0.65 | 0.64 | 0.90 |  | | 0.71 | | -.05-.19 |  | | 1.02 | | + | + | + | -.18 | .001 |  | **DADYS-Screen** | | | |

*Note.* Results are printed in bold, if not matching a priori defined criteria with not considering item difficulties determined based on the population-based sample (see manuscript text, Methods/Analyses); please note positively phrased items were recoded prior to calculating item-inter-correlations, exploratory and confirmatory factor analyses, and IRT analyses; “+” indicates good characteristics according to graphically displayed curves; CFA=confirmatory factor analysis; CCC=Category-Characteristic-Curve; ICC=Item-Characteristic-Curve; IFF=Item Information Function; DIF=Differential Item Functioning, maximal Nagelkerke’s *R*^2^.

*Cronbach*s α (14-item overall scale)=0.91.

**CFA model fit: χ²=388.391; df=77 (p < 0.001); RMSEA=0.072 (90% Confidence interval=0.065-0.079); CFI=0.967.
